# Electrochemical Imaging of Endothelial Permeability Using a Large-Scale Integration-Based Device

**DOI:** 10.1021/acsomega.1c04931

**Published:** 2021-12-01

**Authors:** Kosuke Ino, Hao-Jen Pai, Kaoru Hiramoto, Yoshinobu Utagawa, Yuji Nashimoto, Hitoshi Shiku

**Affiliations:** † Graduate School of Engineering, Tohoku University, 6-6-11 Aramaki-aza Aoba, Aoba-ku, Sendai 980-8579, Japan; ‡ Graduate School of Environmental Studies, Tohoku University, 6-6-11 Aramaki-aza Aoba, Aoba-ku, Sendai 980-8579, Japan; § Frontier Research Institute for Interdisciplinary Sciences, Tohoku University, 6-3 Aramaki-aza Aoba, Aoba-ku, Sendai 980-8578, Japan

## Abstract

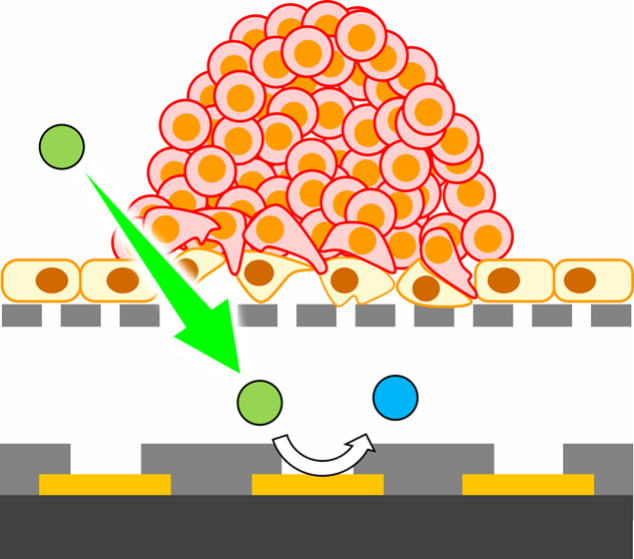

It is important to clarify the transport of biomolecules and chemicals to tissues. Herein, we present an electrochemical imaging method for evaluating the endothelial permeability. In this method, the diffusion of electrochemical tracers, [Fe(CN)_6_]^4–^, through a monolayer of human umbilical vein endothelial cells (HUVECs) was monitored using a large-scale integration-based device containing 400 electrodes. In conventional tracer-based assays, tracers that diffuse through an HUVEC monolayer into another channel are detected. In contrast, the present method does not employ separated channels. In detail, a HUVEC monolayer is immersed in a solution containing [Fe(CN)_6_]^4–^ on the device. As [Fe(CN)_6_]^4–^ is oxidized and consumed at the packed electrodes, [Fe(CN)_6_]^4–^ begins to diffuse through the monolayer from the bulk solution to the electrodes and the obtained currents depend on the endothelial permeability. As a proof-of-concept, the effects of histamine on the monolayer were monitored. Also, an HUVEC monolayer was cocultured with cancer spheroids, and the endothelial permeability was monitored to evaluate the metastasis of the cancer spheroids. Unlike conventional methods, the device can provide spatial information, allowing the interaction between the monolayer and the spheroids to be monitored. The developed method is a promising tool for organs-on-a-chip and drug screening in vitro.

## Introduction

Endothelial and epithelial cell barriers play critical roles in regulating the transport of biomolecules and chemicals to tissues. In particular, vascular endothelial (VE) cell barriers are important because blood vessels are utilized to transport nutrients and drugs. As endogenous mediators such as histamine increase the endothelial permeability, the mechanism by which such molecules affect cell barriers has been investigated.^1^ In addition, interactions between cell barriers and cancers play a crucial role in metastasis. Therefore, for anticancer drug screening in vitro, it is important to prepare a model for endothelial permeability. As a cell barrier model, the transwell system is widely used. In this model, a cell monolayer is prepared on a porous membrane. To assess the barrier function, fluorescent tracers are injected on top of the monolayer, and tracers that diffuse to the bottom are monitored. As an alternative to this assay, the electric resistance of a monolayer of cells, called the transepithelial/endothelial electrical resistance (TEER), can be monitored.^2−4^ Several devices have been reported for TEER measurements, including a piezoelectric biosensor,^5^ a 3D tubular vascular channel,^6^ and a combination of multiple electrode arrays for assessing the cell barrier function and electrical activity.^7^ TEER measurements have been applied for organs-on-a-chip with integrated electrodes^8^ and a blood–brain barrier model consisting of astrocytes and a microvascular endothelial cell monolayer.^9^

In addition to these methods using fluorescent tracers and TEER measurements, electrochemical tracers have recently been used for endothelial permeability assays.^10,11^ In this approach using electrochemical tracers, electrodes are placed near an endothelial monolayer, and electrochemical tracers are loaded on the opposite side of the monolayer. The tracers that diffuse through the monolayer to the electrodes are monitored amperometrically, and the current is used to evaluate the endothelial permeability. This assay is superior to that using fluorescent tracers because it eliminates the need for manual sampling and complex optical instrumentation. Although this electrochemical approach is very attractive for detecting cell barriers, its applications are limited because information about different local areas cannot be obtained owing to the use of a single working electrode. For example, to evaluate interactions between an endothelial monolayer and cancer spheroids, it is necessary to visualize the local cell permeability, which is difficult when using a single sensor. In addition, it is a little complex to separate bottom and top channels. Furthermore, replacing the initial solution with the assay solution containing tracers can lead to chaotic mixing and errors. To solve these problems, we proposed a novel strategy using an electrode array device.

Several types of electrode array devices are available for bioanalysis.^12^ Large-scale integration (LSI)-based devices consisting of electrode arrays have been used for various bioanalysis applications, such as cellular characterization,^13^ label-free imaging of adenosine 5′-triphosphate,^14^ proton imaging of dynamics in the living brain,^15^ tumor cell counting,^16^ bacteria counting,^17^ DNA detection,^18^ glucose detection,^19^ and imaging of metabolites in biofilms.^20,21^ We previously developed an LSI-based amperometric/potentiometric device containing 20 × 20 sensors with a pitch of 250 μm.^22^ Taking advantage of the high-throughput measurements that can be achieved with the LSI device, we have reported the real-time imaging of respiratory activity^23^ and the cell differentiation level of multiple cell aggregates.^24,25^ In addition, this device has been applied for dopamine detection,^26^ plant evaluation,^27^ immunoassays,^28^ cell adhesion evaluation,^29^ and electrochemical motion tracking of microorganisms.^30^ Furthermore, multiple analytes have been simultaneously visualized,^31^ and the developed imaging system has been named electrochemicolor imaging.

In the present study, the LSI-based device was applied to the assessment of endothelial permeability. Briefly, a monolayer of human umbilical vein endothelial cells (HUVECs) is placed in a solution containing electrochemical tracers, [Fe(CN)_6_]^4–^, on the device. Under an applied potential, the electrochemical tracers near the electrode are consumed, which induces diffusion through the monolayer from the bulk solution to the electrodes. Therefore, the current at the electrodes depends on the endothelial permeability. As the strategy does not require the preparation of top and bottom channels, it is superior to conventional transwell systems. In addition, owing to the packed electrode arrays, local information can be obtained in multiple areas. Therefore, this imaging strategy can be used to visualize local endothelial permeability. As a proof-of-concept of this novel electrochemical imaging system, the effects of histamine on an HUVEC monolayer were monitored. Next, as metastatic cancers invade HUVEC monolayers, the proposed strategy was applied to visualize the changes in the endothelial permeability of a coculture model consisting of an HUVEC monolayer and cancer spheroids (HepG2, MCF-7, and MDA-MB-231). Thus, in the present study, we proposed the novel electrochemical imaging system of endothelial permeability using the LSI-based device, and the proof-of-concept was conducted.

## Results and Discussion

### Schemes

**Figure 1:**
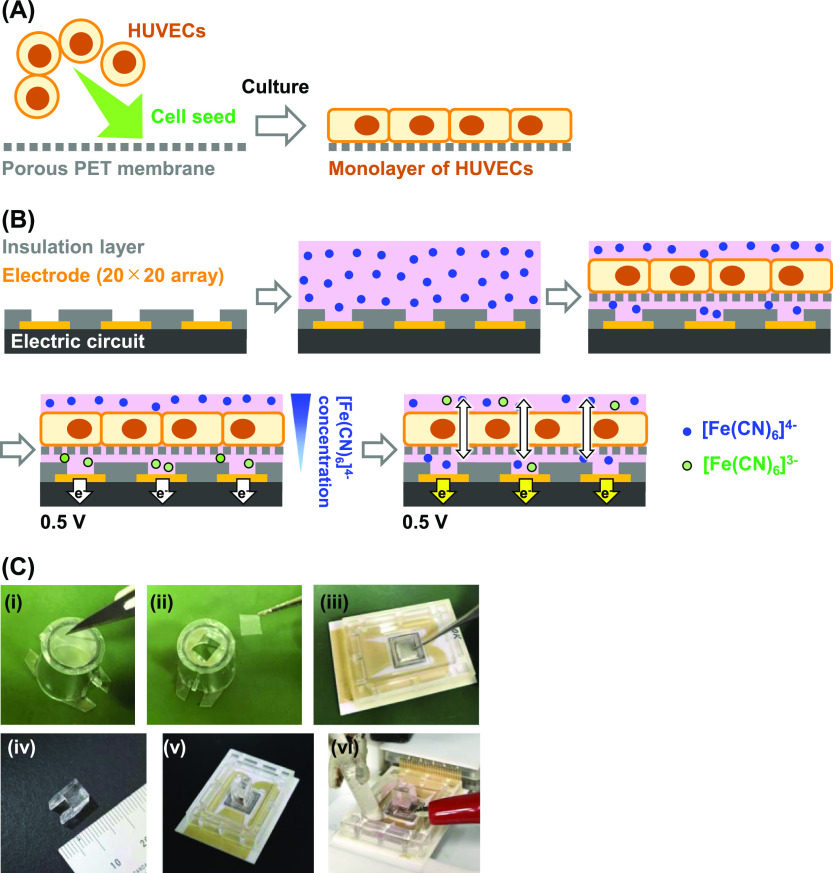
Strategy and process for evaluating endothelial permeability. (A) Fabrication of a HUVEC monolayer on a porous PET membrane. (B) Electrochemical imaging using the LSI-based device. The schematic illustrations are not to scale. (C) Photographs of the detection process: (i,ii) collection of the HUVEC monolayer on the membrane; (iii) placement of the membrane on the LSI-based device; (iv) PDMS frame; (v) pushing of the membrane onto the device surface using the frame; and (vi) electrochemical detection. The reference and counter electrodes are inserted in the assay solution.

Figure 1A,B shows the schemes of preparation of a HUVEC monolayer and detection, respectively. They are described in detail in the Materials and Methods section.

### HUVEC Monolayer

**Figure 2:**
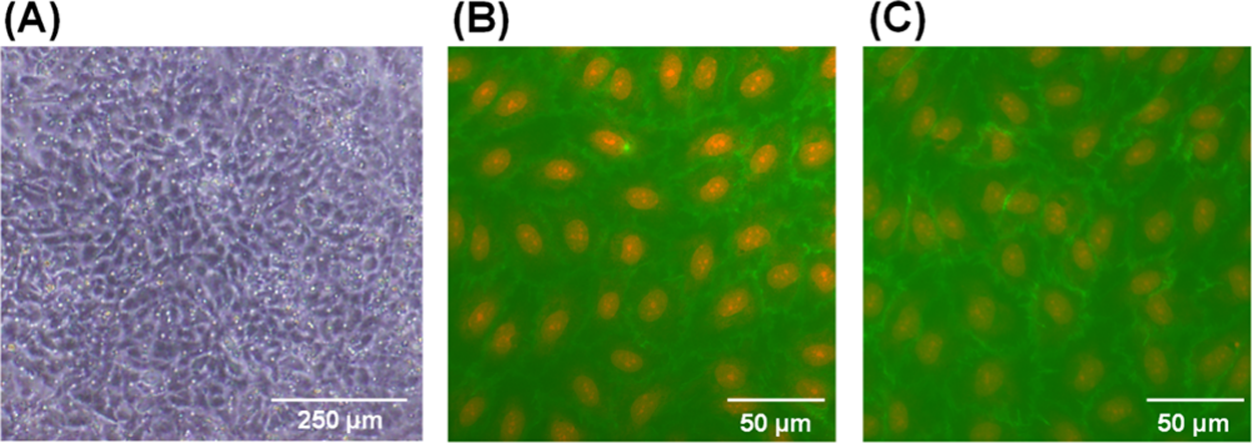
HUVEC monolayer cultured for 7 days. (A) Phase contrast image of the monolayer on the membrane. (B,C) Fluorescence images of VE-cadherin (green) and nuclei (red) in the monolayer on the culture plate: (B) control and (C) after incubation for 30 min in the culture medium containing [Fe(CN)_6_]^4–^.

HUVECs were attached to a collagen-coated membrane and formed a monolayer, which remained after culturing for 7 days (Figure 2A). In the phase contrast image, the microholes in the membrane could also be observed. Although green fluorescent protein (GFP)-expressing HUVECs were used, the cells could be observed using phase-contrast imaging owning to the high transparency of the membrane. Therefore, a discussion of cell imaging using a GFP is omitted from this paper.

During electrochemical imaging, the monolayer was exposed to [Fe(CN)_6_]^4–^ for several minutes. Therefore, the effect of [Fe(CN)_6_]^4–^ on VE-cadherin at intercellular junctions was investigated. Immunostaining showed that VE-cadherin was retained after culturing for 30 min in the medium containing 5 mM [Fe(CN)_6_]^4–^ (Figure 2B,C), indicating that the toxic influence of [Fe(CN)_6_]^4–^ is neglectable during the measurements.

### Amperometric Measurements of Endothelial Permeability

**Figure 3:**
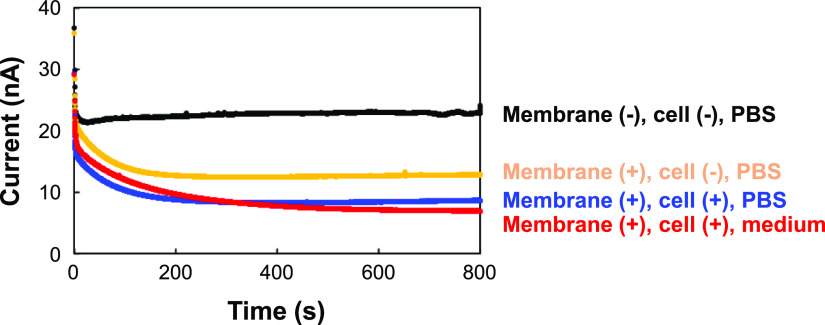
Chronoamperometry for determining cell permeability using the LSI device. Amperograms in PBS (without the membrane, with the membrane but without the HUVEC monolayer, and with the HUVEC monolayer on the membrane) and in the medium containing [Fe(CN)_6_]^4–^ (with the HUVEC monolayer on the membrane).

Figure 3 shows the amperograms obtained using the LSI device under various conditions. First, using phosphate-buffered saline (PBS) containing [Fe(CN)_6_]^4–^, steady currents of approximately 22 nA were obtained in the absence of the membrane. Assuming a diffusion coefficient of 6.5 × 10^–10^ m^2^/s for [Fe(CN)_6_]^4–^,^32^ the theoretical current for a single electrode^33,34^ is approximately 19 nA, which is similar to the experimental value. In contrast, when the sensing area was covered with a collage-coated membrane, the currents decreased to approximately 13 nA, indicating that the diffusion of [Fe(CN)_6_]^4–^ was blocked by the membrane. According to results of a current simulation, the gap between the device surface and the membrane is roughly estimated to be 10 μm (Figure S1). Thus, the current values are determined by the diffusion of [Fe(CN)_6_]^4–^ from the bulk solution through the pores of the membrane to the electrodes. When an HUVEC monolayer was prepared on the membrane, the currents decreased again to 6–8 nA because the diffusion of [Fe(CN)_6_]^4–^ was further blocked by the HUVEC monolayer. These results indicate that the change in current can be used to evaluate the endothelial permeability. Next, to provide better conditions for the cells, a cell culture medium [endothelial cell growth medium 2 (ECGM2)] was used instead of PBS. Similar currents were obtained in both ECGM2 and PBS, indicating that the effects of the cell culture medium on the electrochemical reaction of 5 mM [Fe(CN)_6_]^4–^ are neglectable. Therefore, ECGM2 containing 5 mM [Fe(CN)_6_]^4–^ was used in all subsequent experiments.

Thus, a concentration gradient formed by the consumption of [Fe(CN)_6_]^4–^ in an electrochemical reaction can be used as a driving force for diffusion, resulting in the successful detection of endothelial permeability. As a further proof-of-concept, the influences of histamine and cancer spheroids on the endothelial permeability were monitored.

### Electrochemical Imaging of Endothelial Permeability of HUVEC Monolayers Stimulated with Histamine

**Figure 4:**
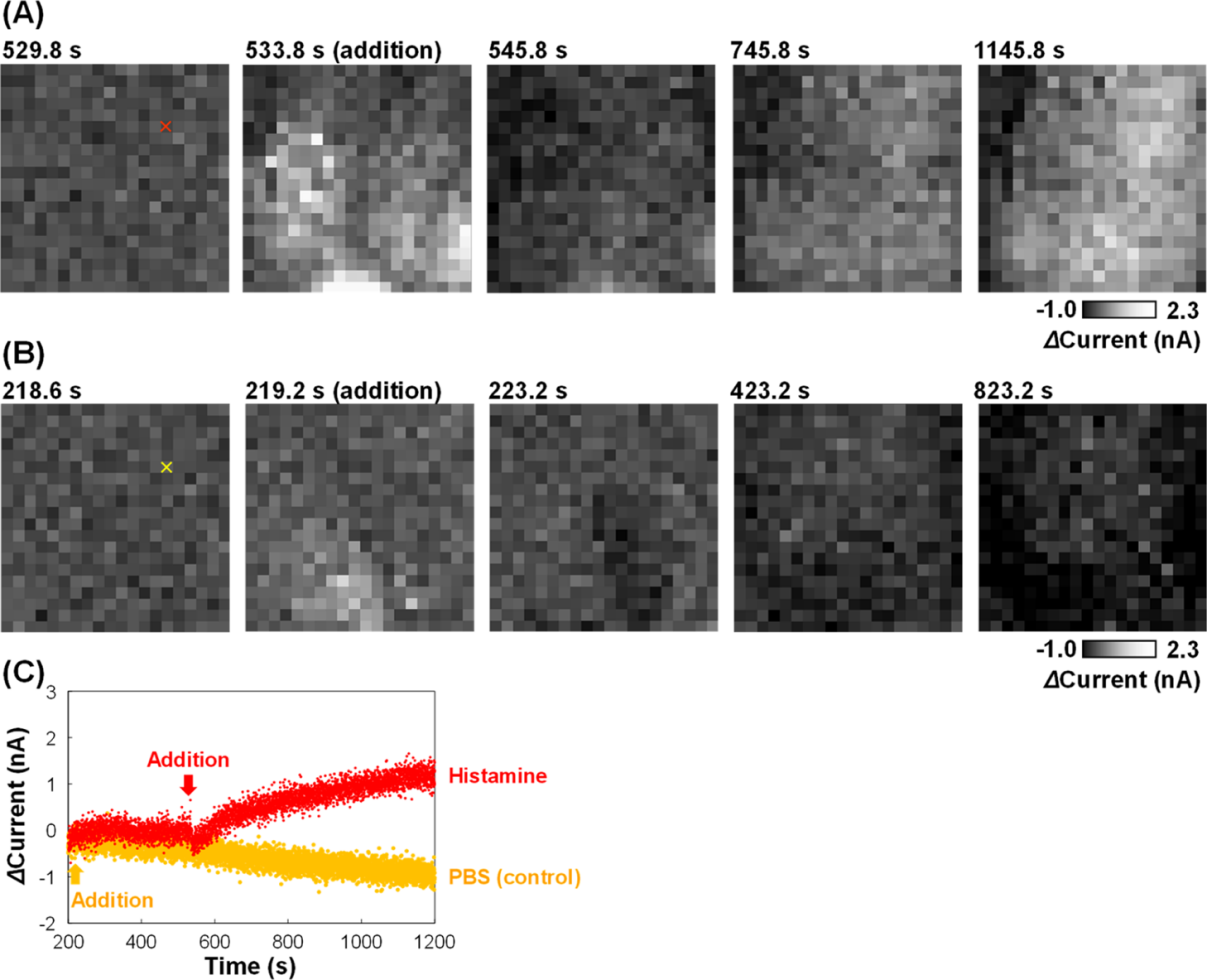
Electrochemical imaging of the HUVEC monolayer on the membrane after the addition of histamine. (A) Electrochemical images following histamine addition (normalized by subtraction of the image at 528 s). (B) Control images following PBS addition (normalized by subtraction of the image at 218.4 s). (C) Chronoamperograms from the sensors marked with red and yellow “x” in (A,B), respectively.

The binding of histamine to receptors on endothelial cell surfaces opens calcium channels, causing cytoskeleton constriction and damage to barrier functions. Figure 4 shows the electrochemical images of the endothelial permeability of an HUVEC monolayer. The addition of a histamine solution at approximately 528 s caused the currents to increase rapidly owing to the mixing of the solution on the membrane (Figure 4A). However, within 10–20 s, the currents returned to the initial value because mixing was complete. Once stable, the current increased gradually over a few minutes, indicating that the endothelial permeability increased following cell damage by histamine. Han et al.^5^ reported that histamine influences the permeability of a cell monolayer within a few minutes to 0.5 h, which is similar to the response time observed in the present study. In contrast, when PBS solution was added as a control, the current values did not increase (Figure 4B). The chronoamperograms at the sensors clearly showed the effect of the histamine (Figure 4C). Although biomolecules from the cells due to the stimulation could be reacted at 0.5 V, the redox current at the 40 μm disk electrode increased by more than 1 nA and persisted for a long time, indicating that the redox currents were mainly derived from 5 mM [Fe(CN)_6_]^4–^ rather than biomolecules from the cells across the porous membrane. Thus, the change in endothelial permeability caused by histamine was successfully monitored using the proposed electrochemical imaging strategy. Although the effects of varying the histamine concentration have not been explored, these results provide a proof-of-concept.

### Electrochemical Imaging of Endothelial Permeability of HepG2 Spheroids

**Figure 5:**
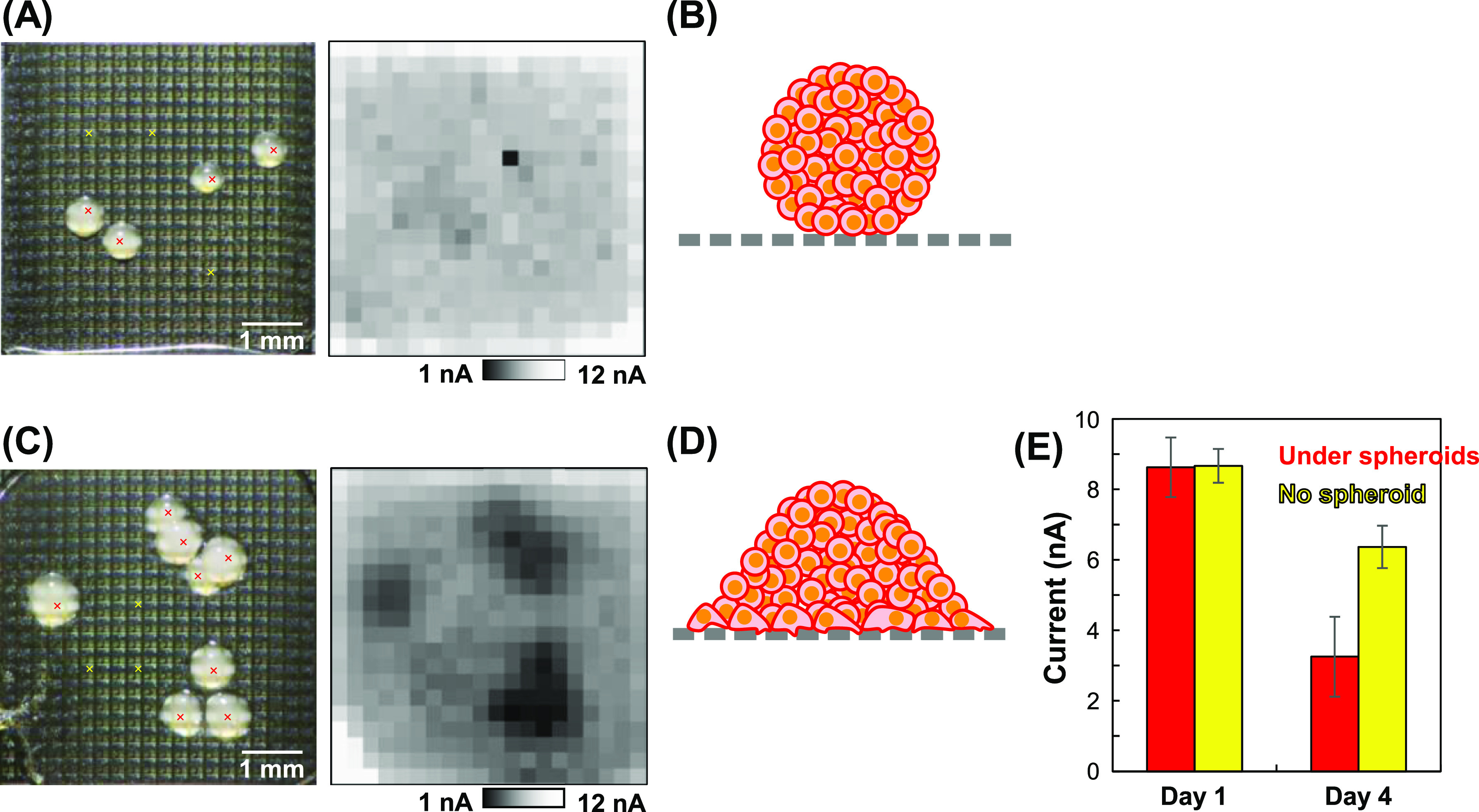
Electrochemical imaging of HepG2 spheroids on the membrane. (A,B) Day 1 and (C,D) day 4. (A,C) Optical (left) and electrochemical images obtained at 299.8 s (right). (B,D) Illustrations of the spheroids on the membranes. The illustrations are not to scale. (E) Currents from the sensors indicated by red and yellow x in (A,C).

As a control for the coculture model, HepG2 spheroids on a membrane without an HUVEC monolayer were electrochemically imaged. Figure 5A,B shows the results after culturing for 1 day, whereas Figure 5C,D shows the results after culturing for 4 days. Figure 5E shows the current values in areas under the spheroids and in those without spheroids. In the areas without spheroids, after culturing for 1 day, the [Fe(CN)_6_]^4–^ oxidation currents decreased to approximately 9 nA (from 13 nA on day 0, Figure 3) with a further decrease to approximately 6 nA observed after culturing for 4 days. These results indicate that some of the pores in the membrane were filled with components of the culture medium even in the areas without cells. Although a single pixel in Figure 5A has a significantly lower current, this might not be derived from cells but might be noise due to the defective sensor.

Although the currents under the spheroids decreased slightly after culturing for 1 day (Figure 5A), the currents were similar to those in the areas without the spheroids (Figure 5E), indicating that the spheroids were weakly adhered to the membrane (Figures 5B and S2A). In contrast, the oxidation currents under the spheroids decreased dramatically to approximately 3 nA after culturing for 4 days (Figure 5C,E), suggesting that the spheroids strongly were adhered to the membrane and the membrane pores were filled with cells (Figures 5D and S2B). In addition, the currents decreased in areas where the cells proliferated and formed a monolayer (Figure 5C,D). According to these results, cell adhesion on the membrane also affects the electrochemical signal.

### Electrochemical Imaging of Endothelial Permeability of HUVEC Monolayers Cocultured with HepG2, MCF-7, or MDA-MB-231 Spheroids

**Figure 6:**
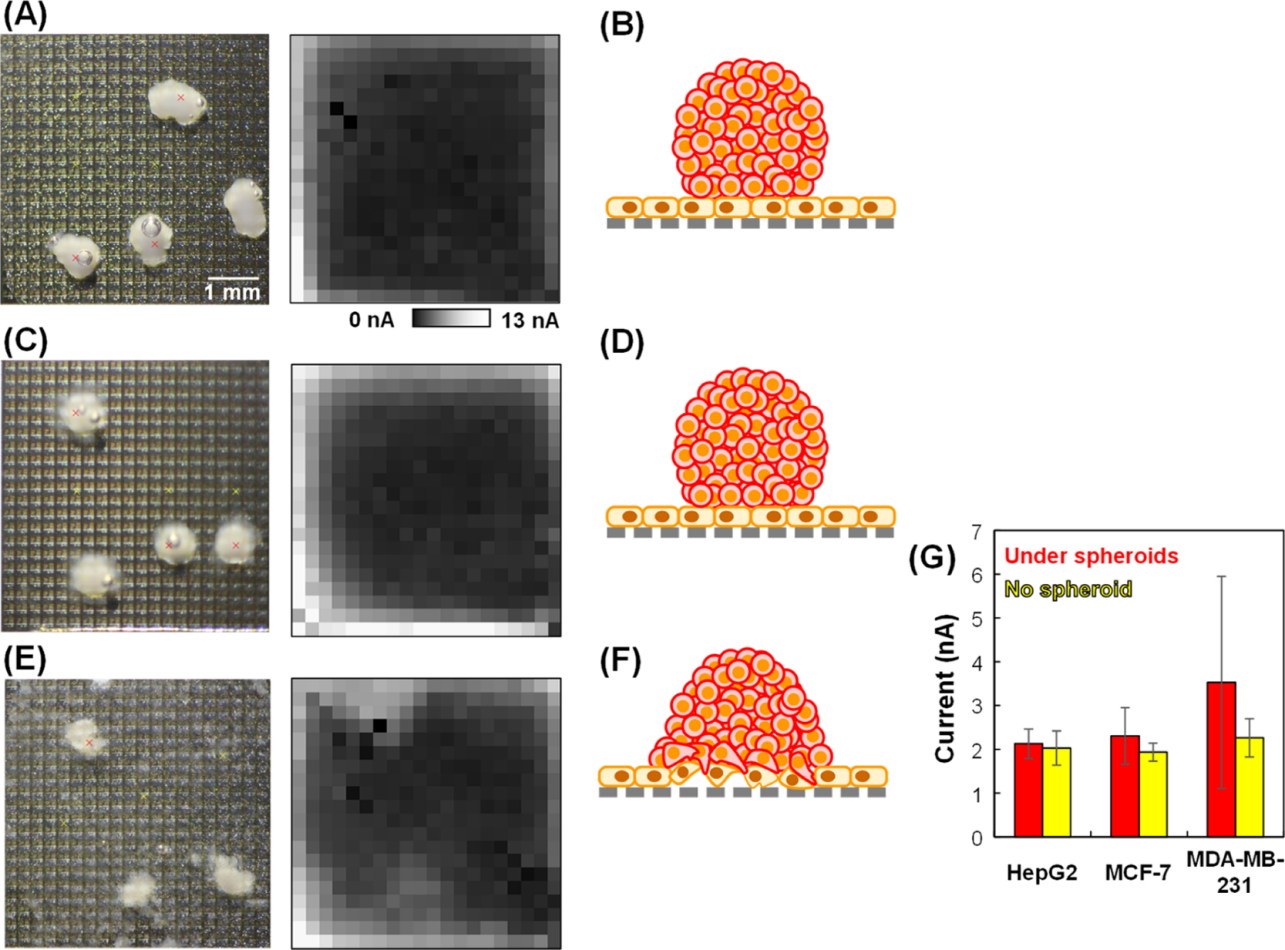
Electrochemical imaging of cocultured cancer spheroids and HUVEC monolayers. (A,B) HepG2 spheroids, (C,D) MCF-7 spheroids, and (E,F) MDA-MB-231 spheroids. (A,C,E) Optical and electrochemical images obtained at 599.8 s. (B,D,F) Illustrations of spheroid cross sections. The illustrations are not to scale. (G) Currents from the sensors indicated by red and yellow x in (A,C).

As a model for analyzing the interactions of a tumor with blood vessel walls, tumor spheroids were cultured on endothelial cell monolayers.^35^ Once attached to a blood vessel, tumor cells can breach or invade the endothelial-based lamina to reach the extracellular connective tissue space and establish a secondary tumor. The cells attach and penetrate the junctions during the earliest stage of the invasion process. As this step is crucial for the development of metastases, it is important to investigate these interactions and effects using in vitro models. Tumor metastasis processes depend on several factors such as cell adhesion molecules, direct cell-to-cell interactions, reactive oxygen species (ROS), angiogenic growth factors, receptors, and proteolytic enzymes.^36^ Previously, a coculture model consisting of tumor spheroids and an HUVEC monolayer was applied to evaluate ROS production.^36^ Microfluidic devices have also been applied to coculture spheroids, mammary epithelial cells, and stroma fibroblasts.^37^ In the present study, as a model of metastasis through an endothelial monolayer, cancer spheroids were seeded on HUVEC monolayers. Figure 6A shows optical and electrochemical images of HepG2 spheroids on the HUVEC monolayer. Large currents were observed at the edge of the electrochemical image because the pushing of the monolayer onto the device was not sufficient and [Fe(CN)_6_]^4–^ diffusion also occurred at the edge of the membrane. Therefore, the currents at the edge were not considered in the following discussion. Surprisingly, the currents under the HepG2 spheroids were similar to those in the areas without the spheroids. The result indicates that HepG2 spheroids might not destroy the HUVEC monolayer (Figure 6B). When using MCF-7 spheroids, a slight change in the currents was observed (Figure 6C), but the trend was similar to that with HepG2, indicating that the MCF-7 spheroids might not destroy the monolayer (Figure 6D). In contrast, a large increase in current was observed under the MDA-MB-231 spheroids (Figure 6E), indicating that the HUVEC monolayer was destroyed by these spheroids (Figure 6F,G). As the invasion ability of MDA-MB-231 cells is higher than that of MCF-7 cells,^38^ the HUVEC monolayer was more affected by MDA-MB-231 spheroids in the present study. Nikshoar et al.^39^ performed invasion assays based on impedance measurements using metastatic MDA-MB-231 cells and nonmetastatic MCF-7 cells added to HUVEC monolayers. They reported that the HUVECs retracted due to the invasion of the MDA-MB-231 cells, whereas the MCF-7 cells did not induce any perturbation in the endothelial barrier. These results are similar to those obtained using the electrochemical imaging strategy although spheroids were used instead of single cells in the present study.

When using larger MCF-7 spheroids, the currents under the spheroids increased, indicating that the endothelial permeability of the HUVEC monolayer increased (Figure S3). This behavior differed from that observed using the small spheroids, suggesting that some destruction of the HUVEC monolayer occurred. During culturing, the spheroids block the diffusion of oxygen nutrients from the bulk solution into the monolayers, and the large spheroids might have a larger effect on this process than the small ones.

Thus, the present electrochemical imaging method can be utilized to evaluate cancer metastasis because information about the local areas can be obtained. However, in this study, we did not consider the direct adhesion of the cancer spheroids on the membranes after the destruction of the HUVEC monolayers because this process is very complicated. As the direct adhesion of the spheroids will affect the diffusion of [Fe(CN)_6_]^4–^, these effects should be determined to improve the precision of the analysis.

## Conclusions

We developed an electrochemical imaging method to evaluate the endothelial permeability of HUVEC monolayers. Although the presented results are preliminary, they provide a proof-of-concept. As diffusion of the electrochemical tracers is induced by the electrochemical reaction at the packed electrodes, separated channels are unnecessary, which is an advantage over conventional methods. Moreover, the use of an electrode array allowed information about local areas to be obtained, resulting in the successful imaging and evaluation of the metastasis of cancer spheroids on HUVEC monolayers. However, in the current system, the HUVEC monolayers were pushed onto the sensor surface by hand, resulting in large errors, and the membrane with the HUVEC monolayer was cut by hand, which is not a sophisticated approach. To solve these problems, a culture system is needed although it will complicate the detection system.

In the present study, cancer spheroids were used as primary tumor models. Cancer cells in primary tumors, such as spheroids, invade a vascular lumen from the tissues via an endothelial monolayer. Herein, the cancer spheroids and endothelial monolayer were cultured on the same side of the membrane because the co-culture model was easy to prepare although the configuration did not match the in vivo configuration. Therefore, the configuration should be modified in future studies. Although cells were only cultured on one side of the membrane in this study, cells can also be cultured on the other side. Therefore, the present detection system can be applied to develop further complex models such as a blood–brain barrier model. Furthermore, as the detection system can provide spatial information, it can be applied to visualize interactions between several kinds of cells.

## Materials and Methods

### Electrochemical Imaging Strategy for Determining Endothelial Permeability

First, an HUVEC monolayer is prepared on a porous polyethylene terephthalate (PET) membrane (Figure 1A). Next, the monolayer is placed in a solution containing [Fe(CN)_6_]^4–^ on the LSI device and pushed onto the surface of the device (Figure 1B). Under an applied potential of 0.5 V, [Fe(CN)_6_]^4–^ is oxidized to [Fe(CN)_6_]^3–^, which decreases the [Fe(CN)_6_]^4–^ concentration near the device surface to nearly zero because the packed electrode array operates in the microspace under the monolayer. Therefore, a [Fe(CN)_6_]^4–^ concentration gradient is formed between the solutions above and below the monolayer, which induces [Fe(CN)_6_]^4–^ diffusion. When the endothelial permeability is high, [Fe(CN)_6_]^4–^ mainly diffuses to the electrodes through the monolayer, resulting in large oxidation currents of [Fe(CN)_6_]^4–^ at the electrodes. Thus, the current value can be used as an indicator of the endothelial permeability. Compared with a large single electrode, microelectrodes in an array offer the advantage of sensitivity. Therefore, an LSI-based device is used as a packed electrode array. In addition, electrochemical imaging using such an array of individual working electrodes allows the local endothelial permeability to be evaluated in multiple areas.

### Device Fabrication and Detection System

The device fabrication process is described in our previous papers.^22,31^ Briefly, 20 × 20 Pt working electrodes were prepared on an LSI chip and then covered with SU-8 microwells (diameter: 40 μm, depth: 5 μm). Therefore, the individual working electrodes consist of Pt disk electrodes with a diameter of 40 μm. The pitch of the sensors was 250 μm, and the sensing area was approximately 5 × 5 mm^2^. Potential control and data acquisition were performed using a program written in LabVIEW (National Instruments, USA). Detection instruments and systems were partially developed by Japan Aviation Electronics Industry, Limited (Japan) and purchased from Senschip (Japan). Other details, including electronic circuits, are described in our previous papers.^22,31^

### Cell Cultures

GFP-expressing HUVECs were purchased from Angio-Proteomie (USA). The cells were cultured in ECGM2 (Promo Cell, Germany) containing 1% penicillin/streptomycin (PS, Gibco, USA). HepG2 (hepatocellular carcinoma cell line, ATCC, USA) and MDA-MB-231 (breast cancer cell line, ATCC) were cultured in Dulbecco’s modified Eagle’s medium (4.5 g/L glucose, Gibco) containing 10% fetal bovine serum (FBS, Gibco) and 1% PS (Gibco). MCF-7 (Tohoku University) was cultured in RPMI1640 (Gibco) containing 10% FBS and 1% PS. All cells were cultured at 37 °C in a humidified atmosphere containing 5% CO_2_. Phase-contrast images of the cells were captured using a microscope (Olympus IX71, Japan).

### Preparation of HUVEC Monolayers on Porous Membranes

Rat tail collagen type I (Corning, USA) was diluted to 0.0574 μg/mL using PBS (Nacalai Tesque, Japan), and 0.25 mL of this solution was placed in each well of a 12-well cell culture insert (Corning) consisting of a PET membrane (pore size: 8 μm, pore density: 1 × 10^5^ pores/cm^2^, thickness: 10.5 μm). The culture insert was incubated at 37 °C for 30 min. After removing the solution, the culture insert was washed with PBS. Then, HUVECs (3.0 × 10^4^ cells/cm^2^) were seeded in the culture insert and cultured in ECGM2 for 7 days to prepare the HUVEC monolayers (Figure 1A). The medium was changed every 2 days. After culturing, the membranes were cut into pieces of approximately 6 × 6 mm^2^ for electrochemical assays.

### Immunostaining of VE-Cadherin in HUVEC Monolayers

To investigate the effects of [Fe(CN)_6_]^4–^ on HUVEC monolayers, VE-cadherin in the HUVEC monolayers was stained. First, HUVECs were cultured in a 24-well culture plate (Corning) for 7 days to prepare HUVEC monolayers. The monolayers were washed with PBS three times and then immersed in PBS containing 4% paraformaldehyde (Fujifilm Wako Pure Chemical Co., Japan) for 15 min. Next, after washing with PBS three times, the monolayers were immersed in 0.1% Triton X-100 (Sigma-Aldrich Japan, Japan) for 15 min. After removing Triton X-100, the monolayers were immersed in PBS containing 1% bovine serum albumin (Sigma-Aldrich Japan) and rabbit anti VE-cadherin antibody (1:500 dilution, Cell Signaling Technology, USA). After incubating overnight at 4 °C, the solution was replaced with PBS containing a secondary antibody, Alexa Fluor 647 conjugated goat anti-rabbit IgG (1:1000 dilution, Thermo Fisher Scientific, USA), and 45 μM propidium iodide (PI, double staining kit, Dojindo, Japan). The monolayers were incubated overnight at 4 °C and then observed under a fluorescence microscope (Leica DMi8, Leica, Germany).

### Cocultures of Cancer Spheroids and HUVEC Monolayers

A 96 U well plate (Sumitomo Bakelite Co., Japan) was used to prepare the cancer spheroids. First, 200 μL of a HepG2, MCF-7, or MDA-MB-231 suspension was introduced into each well (2000 cells/well) and then cultured for 4 days to form spheroids. The spheroids were carefully transferred onto the HUVEC monolayer that had been cultured for 7 days on the membrane. The coculture model was further cultured for 7 days. After culturing, the membrane was cut into pieces of approximately 6 × 6 mm^2^ for electrochemical assays.

### Electrochemical Imaging of Endothelial Permeability

Figure 1C shows photographs of the detection process. After preparing the HUVEC monolayers on the membranes, a piece of the membrane was immersed in ECGM2 containing 5 mM [Fe(CN)_6_]^4–^ on the device. The membrane was pushed onto the device surface using a polydimethylsiloxane frame (Dow Corning Toray, Japan). Then, Ag/AgCl (sat. KCl) reference and Pt counter electrodes were inserted into the solution. A voltage of 0.5 V was applied to the 400 working electrodes and the current from each electrode was obtained every 200 ms. The current values were converted into colors to construct electrochemical images consisting of 20 × 20 pixels.

For the assays using histamine, 45 μL of 0.333 mM histamine (Merck, Germany) was added into ECGM2 containing 5 mM [Fe(CN)_6_]^4–^ (final histamine concentration: 10 μM). The change in the current values was monitored in real time..

## Supporting Information Available

Current simulation of redox compounds, phase-contrast images of HepG2 spheroids, and electrochemical imaging of HUVEC monolayers (PDF)
