# Supplementary material for: Electrochemical Imaging of Endothelial Permeability Using a Large-Scale Integration-Based Device
Source: ACS Omega. 2021 Dec 1;6(51):35476–83. doi: 10.1021/acsomega.1c04931 (PMC8717544; doi:10.1021/acsomega.1c04931)
Supplement: Supplementary file 1 — ao1c04931_si_001.pdf [file ao1c04931_si_001.pdf]

Supporting Information for

Electrochemical imaging of endothelial permeability using a large-scale integration-based device

Kosuke Ino[1,*], Hao-Jen Pai[2], Kaoru Hiramoto[2], Yoshinobu Utagawa[2], Yuji Nashimoto[1,3], Hitoshi Shiku[1,*]

[1] Graduate School of Engineering, Tohoku University, 6-6-11 Aramaki-aza Aoba, Aoba-ku, Sendai 980-8579, Japan

[2] Graduate School of Environmental Studies, Tohoku University, 6-6-11 Aramaki-aza Aoba, Aoba-ku, Sendai 980-8579, Japan

[3] Frontier Research Institute for Interdisciplinary Sciences, Tohoku University, 6-3 Aramaki-aza Aoba, Aoba-ku, Sendai 980-8578, Japan

Corresponding authors: Kosuke Ino (kosuke.ino@tohoku.ac.jp) and Hitoshi Shiku (hitoshi.shiku.c3@tohoku.ac.jp)

Table of Contents

- Figure S1
- Figure S2
- Figure S3

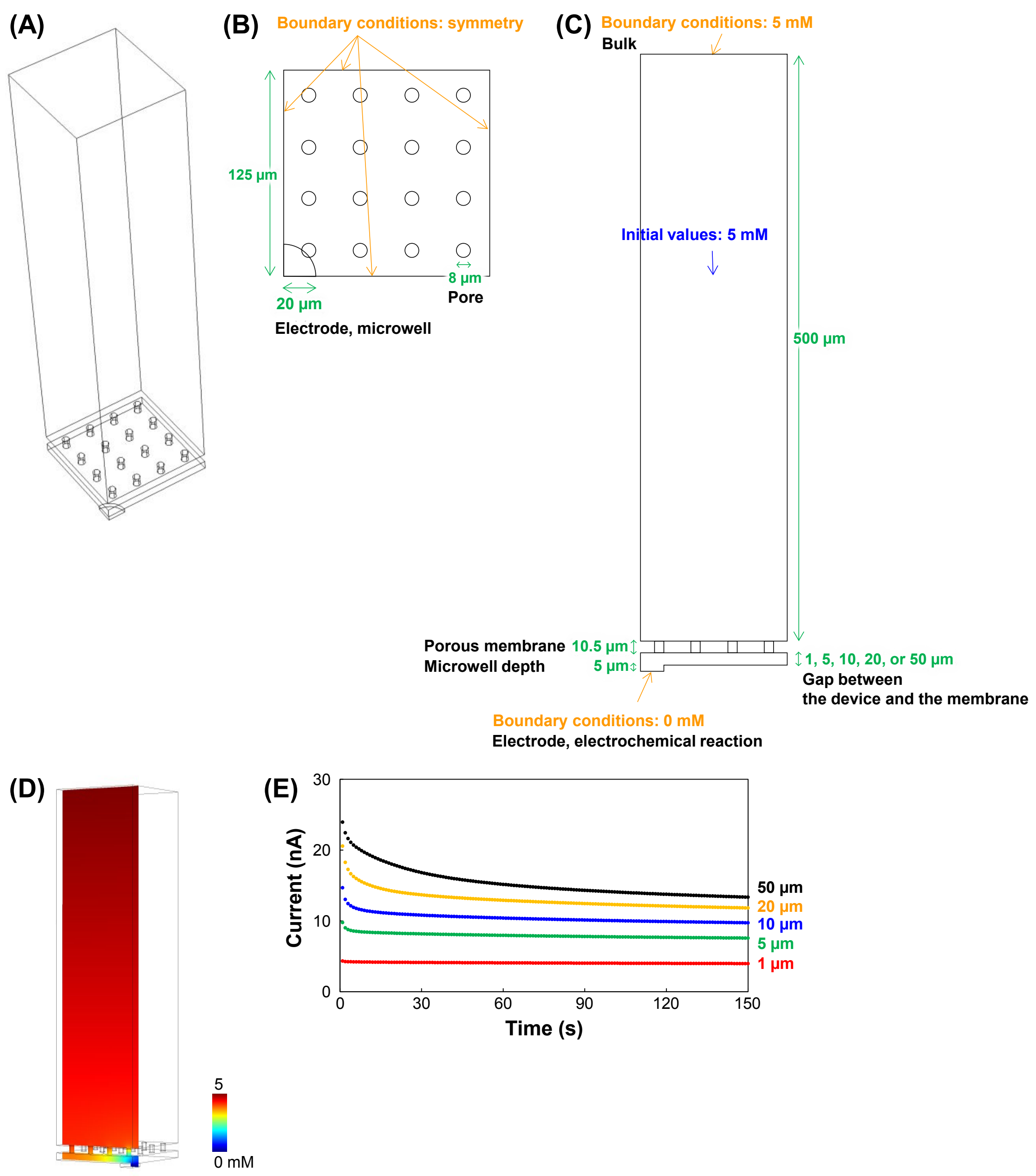

Figure S1

Current simulation for redox compound diffusion using COMSOL Multiphysics (ver. 5.4, Comsol, Inc., USA). (A) Overall, (B) top, and (C) cross-sectional views of a 3D model consisting of a porous membrane, a microwell, and a working electrode. Boundary conditions and initial values are shown in the illustrations. To simply the model, symmetry was set for the boundary conditions. $[Fe(CN)_6]^{4-}$ is filled in the model, and its initial value was set to 5 mM. The diffusion coefficient was set to 7×10^{-10} m^2/s. Since the sufficient potential is applied to oxidize $[Fe(CN)_6]^{4-}$ to $[Fe(CN)_6]^{3-}$, the boundary condition of the electrode was set to 0 mM. The flux is set to zero at the insulation surface

and the membrane. In the simulation, the electrochemical system is a reversible one-electron reaction. The concentration gradient on the electrode, the Faraday constant, the diffusion coefficient, and a unit area were used to calculate the oxidation currents of $[Fe(CN)_6]^{4-}$. (D) Profile of $[Fe(CN)_6]^{4-}$ concentration at 150 s when the gap is 10 μm. (E) Amperograms when the gap is 1, 5, 10, 20, and 50 μm.

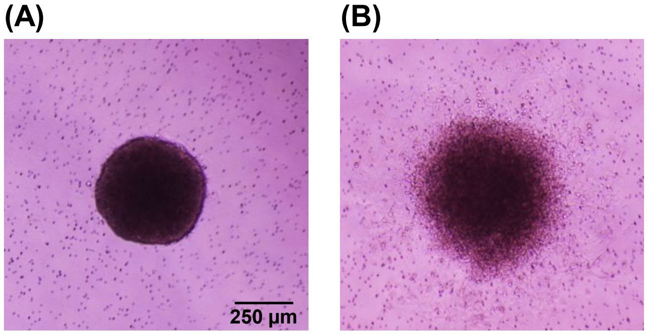

Figure S2

Phase-contrast images of the HepG2 spheroids on the membrane. (A) Day 1 and (B) day 4.

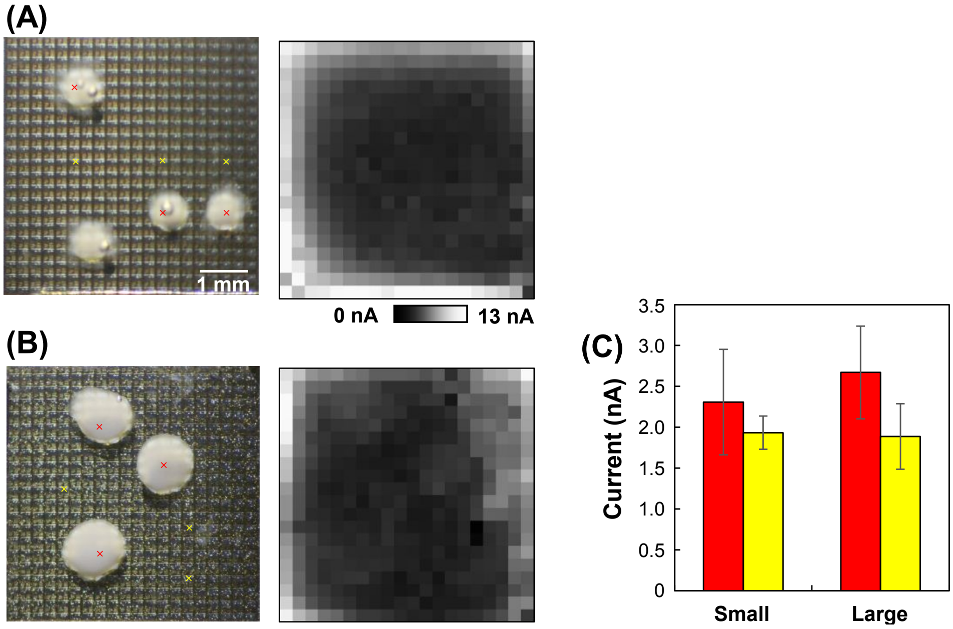

Figure S3

Electrochemical imaging of HUVEC monolayers with large MCF-7 spheroids. (A) Day 1 and (B) day 4. (A, B) Optical (left) and electrochemical images obtained at 299.8 s (right). (C) Currents from the sensors indicated by red and yellow x in (A) and (B). (A) is reproduced in Fig. 6.
